# Living in the “Bubble”: Athletes' Psychological Profile During the Sambo World Championship

**DOI:** 10.3389/fpsyg.2021.657652

**Published:** 2021-05-26

**Authors:** Ambra Gentile, Tatjana Trivic, Antonino Bianco, Nemanja Lakicevic, Flavia Figlioli, Roberto Roklicer, Sergey Eliseev, Sergey Tabakov, Nebojsa Maksimovic, Patrik Drid

**Affiliations:** ^1^Sport and Exercise Sciences Research Unit, University of Palermo, Palermo, Italy; ^2^Faculty of Sport and Physical Education, University of Novi Sad, Novi Sad, Serbia; ^3^Russian State University of Physical Education, Sports, Youth and Tourism, Moscow, Russia

**Keywords:** combat sport, martial arts, stress, performance, COVID-19, quarantine

## Abstract

**Background:** The COVID-19 pandemic has changed the way we conduct daily life, as well as sports training and sports competitions. Given the stress produced by COVID-19, and the “bubble” safety measures for the World Sambo Championship, held in Novi Sad, from the 6th to the 8th of November, 2020, athletes might have experienced more stress than athletes normally would in non-pandemic conditions. Therefore, the current study aimed to create a psychological profile of sambo athletes participating in the Sambo World Championship and living in this condition.

**Methods:** One-hundred-fifteen participants took part in the study, completing the Profile of Mood Scale (POMS), the Pittsburg Sleep Quality Index, the Perceived Stress Scale (PSS), and the Fear of COVID-19 Scale. A mediation model with Fear of COVID-19 predicting both stress level directly and stress level through mood disturbance was hypothesized. Gender differences were evaluated through *t*-test.

**Results:** The results showed that the sample presented higher levels of stress but no problems in sleeping. In particular, data analysis confirmed an indirect effect of Fear of COVID on Perceived stress through mood disturbance (β = 0.14, *Z* = 2.80, and *p* = 0.005), but did not have a significant impact on the direct effect (β = −0.04, *Z* = −0.48, and *p* = 0.63). Gender differences emerged in the perceived stress level (*t* = −2.86, *df* = 114, and *p* = 0.005) and daytime dysfunction (*t* = −2.52, *df* = 114, and *p* = 0.01) where females scored higher than males for both aspects.

**Conclusion:** The athletes participating to the World Sambo Championship experienced stress levels determined by the mood disturbance produced by the fear of the COVID-19 pandemic. Female athletes were more stressed and showed higher daytime dysfunction. The findings of the current study are useful to understand the psychological profile of the athletes competing in the “bubble” conditions during COVID-19 pandemic.

## Introduction

The COVID-19 pandemic outbreak dramatically changed how we conduct our daily lives (Pišot et al., 2020; Serafini et al., 2020). From March 2020, countries worldwide are facing movement restrictions measures (e.g., quarantine) with the aim to limit the viral spread, but at the same time, these preventative measures have worsened people's mental health (Brooks et al., 2020). For instance, a meta-analysis has investigated this negative impact across 17 studies and found that the most prevalent reaction was anxiety, followed by depression and stress (Salari et al., 2020).

Previous studies have shown that the fear generated in response to the COVID-19 situation leads to increase in depression, anxiety and stress, impacting people's positive affect (Bakioglu et al., 2020). Specifically, the fear in response to the pandemic has raised anxiety levels and fear of the unknown, both in healthy individuals and individuals with pre-existing mental issues (Shigemura et al., 2020).

The fear related to the pandemic, including developing COVID-19 infection, also impacted mood balance (Usher et al., 2020), and stress (Makarowski et al., 2020). Furthermore, a low sleep quality, characterized by insomnia, nightmares, and sleep apnea, was strongly associated to mood disorders, particularly anxiety.

Considering gender differences, current data has shown that COVID-19 pandemic had a worse impact on women than in men for two main reasons: women were more likely to permanently lose their job than men (Dang and Nguyen, 2020) with females showing higher tendency of displaying more post-traumatic stress symptoms (Liu et al., 2020), and higher levels of anxiety, depression and acute stress than males (Garcia-Fernandez et al., 2021).

Concerning the domain of sports, the athletes underwent severe modifications in how they train, compete, and socialize. Depending on the country and specific national laws, many athletes had to cease their regular training regimen during various periods of the COVID-19 pandemic (Paoli and Musumeci, 2020), especially those involved in team sports. In this perspective, a world championship competition represents a huge source of stress, characterized by fear of failure, feelings of inadequacy, external control, and social evaluation (Gould et al., 1983).

Within this scenario, the current circumstances caused by the ongoing COVID-19 pandemic can likely aggravate already delicate psychological status of sambo athletes approaching the competition. Specifically, the sambo World Championship, held in Novi Sad from the 6th to the 8th of November 2020, was organized following a particular set of procedures named “the bubble,” a sort of quarantined sport competition whereby athletes were only able to spend time in a hotel or sports arena.

Taking into account “bubble” atmosphere seen at many recent athletic events, specific competitive conditions caused by the pandemic are a potent stimulus Fear of COVID-19 in mood regulation disruption, and in turn, potential elevated levels of stress and insufficient sleep quantity and quality that can ultimately result in impaired health and performance decrements. Therefore, this study aimed to examine the psychological profiles of athletes participating in the World Sambo Championship, with a particular emphasis on gender differences. In addition, to understand the impact of COVID-19 on the perception of stress in the athletes, a mediation model was hypothesized, where the Fear of COVID determines mood disturbance, which in turns produces high levels of perceived stress in the athletes.

Moreover, based on the previous findings we hypothesized that females should report higher levels of fear of COVID-19, mood disturbance, and stress levels compared to the male counterparts.

## Materials and Methods

### Participants

The initial sample originally consisted of 209 participants, but 94 were excluded as they did not complete all the measures. The final sample consisted of 115 participants (mean age = 22.3 years, SD = ±5.51 years), of which 78 were males (67.8%) and 37 were females (32.2%). The mean height was 171 cm (SD = ±10 cm) and the mean weight was 72.02 kg (SD = ±18.54). The average years of sambo experience was 11.26 years (SD = ±6.00 years). Data collection took place in the sports hall during the World Sambo Championship in Novi Sad, Serbia taking place from the 6th to the 8th of November 2020.

Athletes were asked to participate in the experiment prior to warm-up before fights or after they were eliminated from competing further. A team of several experienced researchers were responsible for handing out the questionnaires, instructing athletes how to respond, and making sure that questionnaires were fully completed. All questionnaires were translated and offered in Serbian, Russian, English, and French language. To assist in the data acquisition, a translator who spoke all four languages mentioned above was hired. In case an athlete handed out an obviously incomplete questionnaire, this sample was immediately eliminated.

The study was approved by the Institutional Review Committee of the University of Novi Sad (Ref. No. 46-06-02/2020-1) and was conducted following the principles indicated in the Declaration of Helsinki. All included participants provided an informed consent, and they were told that they were free to give up the fulfilling at any stage without any consequence.

The pre-requisites for each athlete's participation were the negative RT-PCR test (Reverse-Transcriptase-polymerase chain reaction), used to detect the infection Sars-CoV-2, mandatory face masks, regular temperature check, strict safety measures (such as social distancing, mask-wearing, and hand hygiene). Moreover, the transportation was strictly supervised: all the teams used separate busses that were sanitized twice a day and trained in separate sports facilities, such as personal mats that were usable after personal disinfection. When athletes and staff people were not training or competing, they spent their time in the hotel, and they were not supposed to go outside for any reason (further information can be found in Supplementary Material 1).

### Measures

#### Profile of Mood Scale

For the assessment of athletes' mood, the Profile of Mood Scale—Abbreviated Version (Grove and Prapavessis, 1992) was employed. This self-report scale is specifically tested for athletes and consists of 40 adjectives measuring seven dimensions, namely tension (TEN), depression (DEP), anger (ANG), fatigue (FAT), confusion (CON), vigor (VIG), and esteem-related affect (ERA), on a scale going from 0 = not at all to 4 = Extremely. The Total Mood Disturbance (TMD) score was calculated through the formula suggested by the Authors:

$$\begin{array}{l} \text{TMD}=\left[\text{TEN}+\text{DEP}+\text{ANG}+\text{FAT}+\text{CON}\right]-\left[\text{VIG}+\text{ERA}\right] \end{array}$$

According to the Authors' instruction, higher scores indicate higher mood disturbance. For eliminating the negative scores, a constant of 100 was added to the TMD score. The scale revealed a good reliability (α = 0.88).

#### Pittsburg Sleep Quality Index

The Pittsburg Sleep Quality Index—PSQI (Buysse et al., 1989) assesses the subjective quality of sleep retrospectively and considering the previous month through 10 questions. For the last question, the respondent should pose the question to the roommate or bed partner, but this part was excluded for the purpose of our research. The questionnaire consists of seven components (Subjective Sleep Quality, Sleep Latency, Sleep Duration, Habitual Sleep Efficiency, Sleep Disturbances, Use of Sleep Medication, and Daytime Dysfunction), plus a global PSQI score obtained through the sum of the previous dimensions. The global score ranges from 0 to 21, and after five the quality becomes poorer and poorer. The scale revealed a good reliability (α = 0.82).

#### Fear of COVID-19

The Fear of COVID-19 scale (Ahorsu et al., 2020) is a 7-item unidimensional questionnaire assessing the negative feelings derived from the Coronavirus Disease pandemic (COVID-19). The questions are evaluated on a 5-point Likert scale from 1 = Strongly Disagree to 5 = Strongly Agree and concern the physical and psychological reactions when thinking about the pandemic. The scale revealed a good reliability (α = 0.80).

#### Perceived Stress Scale

The Perceived Stress Scale—PSS (Cohen et al., 1994) is a 10-items unidimensional scale measuring the stress appraised in a particular situation. The scale also includes direct questions about the degree of stress currently experienced. The questions are evaluated on a scale ranging from 0 = Never to 4 = Always. The final score is obtained through the sum of the scores attributed to all the questions, where the higher is the score, the higher is the perceived stress. The scale revealed a good reliability (α = 0.76), with a similar value detected in previous studies (Cohen et al., 1988; Baik et al., 2019).

### Data Analysis

Data were analyzed through R software (version 3.2.5). Descriptive statistics were performed on the sociodemographic variables, such as age, height, weight, years of experience, and on the scores of perceived stress, fear of COVID-19, mood state, and sleep quality. Gender differences were also evaluated concerning the overmentioned variables through *t*-test comparison.

**Figure 1:**
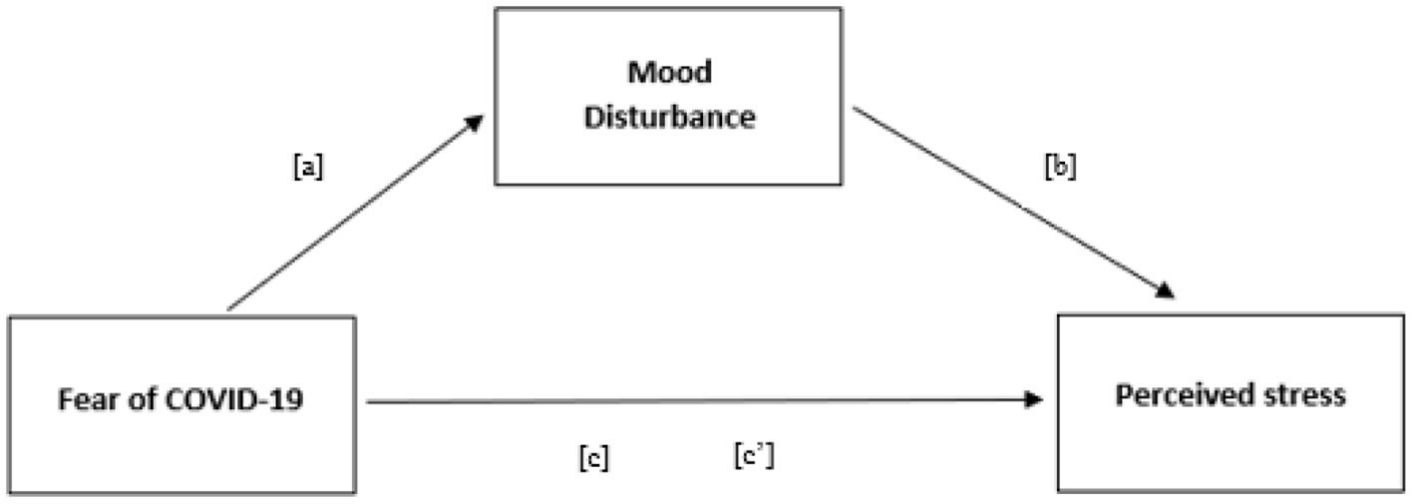
Hypothesized mediation model with Fear of COVID-19 as predictor, mood disturbance as mediator, and perceived stress as outcome for the whole sample.

Since the overall sleep quality was classified as good for the sample, it was excluded from the subsequent analyses. Correlations among fear of COVID-19, perceived stress, and mood disorders were calculated through Pearson's correlation coefficient. Moreover, to assess if the stress experienced by athletes was partially determined by COVID pandemic, a mediation model with direct effect of Fear of COVID-19 on perceived stress [c'], and an indirect effect [c] of Fear of COVID-19 as predictor of mood disturbance [a] and mood disturbance as mediator of perceived stress [b], was evaluated (Figure 1). A direct and an indirect mediation effect from the fear of COVID-19 was hypothesized. Since the sample was not big enough to evaluate the intercorrelations among subdimensions, the model was built only for total scores. The mediation model was performed through the R package *Lavaan* and was conducted following the indications of Baron and Kenny (1986). The alpha level for refusing null hypothesis was set at α = 0.05. The number of female athletes participating to the interview was not enough to test the mediation model distinguished by gender.

## Results

Descriptive statistics of the sample are summarized in Table 1. As mentioned above, the average sleep quality index was 3.54, therefore under 5 that is the cut-off for sleep disorders. Moreover, all the subdimensions were far away from the maximum of each scale that is 3. Therefore, sleep quality was excluded from the subsequent analyses. The sample showed high levels of perceived stress than the normative scores (*t* = 2.96, *df* = 114, and *p* = 0.002).

**Table 1:** Descriptive statistics concerning sleep quality, perceived stress, fear of COVID-19, and mood state.

	**Total**		**Males**		**Females**		
	**Mean**	**SD**	**Mean**	**SD**	**Mean**	**SD**	***t***
**Perceived stress**	15.8	5.69	14.75	5.27	17.92	6.03	−2.87^**^
**Total mood disturbance**	104	21.6	104.43	20.08	101.53	24.83	0.65
Tension	6.29	4.61	6.18	4.34	6.51	5.18	−0.36
Anger	6.07	5.01	6.31	4.71	5.57	5.64	0.74
Depression	6.11	6.12	6.27	5.74	5.76	6.89	0.42
Fatigue	5.26	3.96	5.19	3.67	5.40	4.55	−0.27
Confusion	5.56	3.97	5.69	3.75	5.27	4.43	0.53
Vigor	12.8	4.36	12.59	4.39	13.36	4.30	−0.87
Esteem-related affect	12.0	3.20	12.01	3.38	12.05	2.81	−0.07
**Fear of COVID-19**	16.1	5.01	16.41	5.37	15.38	4.13	1.03
**Sleep quality index**	3.58	2.34	3.53	2.13	3.69	2.77	−0.34
Subjective sleep quality	0.50	0.58	0.51	0.53	0.49	0.69	0.23
Sleep latency	0.92	0.80	0.95	0.82	0.86	0.75	0.53
Sleep duration	0.36	0.91	0.34	1.03	0.40	0.60	−0.36
Habitual sleep efficiency	0.48	0.86	0.40	0.80	0.65	0.98	−1.36
Sleep disturbances	1.09	0.65	1.12	0.69	1.03	0.56	0.74
Use of sleep medication	0.22	0.56	0.18	0.45	0.29	0.74	−0.89
Daytime dysfunction	0.60	0.63	0.50	0.55	0.81	0.74	−2.52^*^

* *p < 0.05*;

** *p < 0.01*.

Concerning gender differences, female athletes reported higher stress levels (*t* = −2.86, *df* = 114, and *p* = 0.005) and higher daytime dysfunction (*t* = −2.52, *df* = 114, and *p* = 0.01) compared to male athletes. No other gender differences emerged.

**Figure 2:**
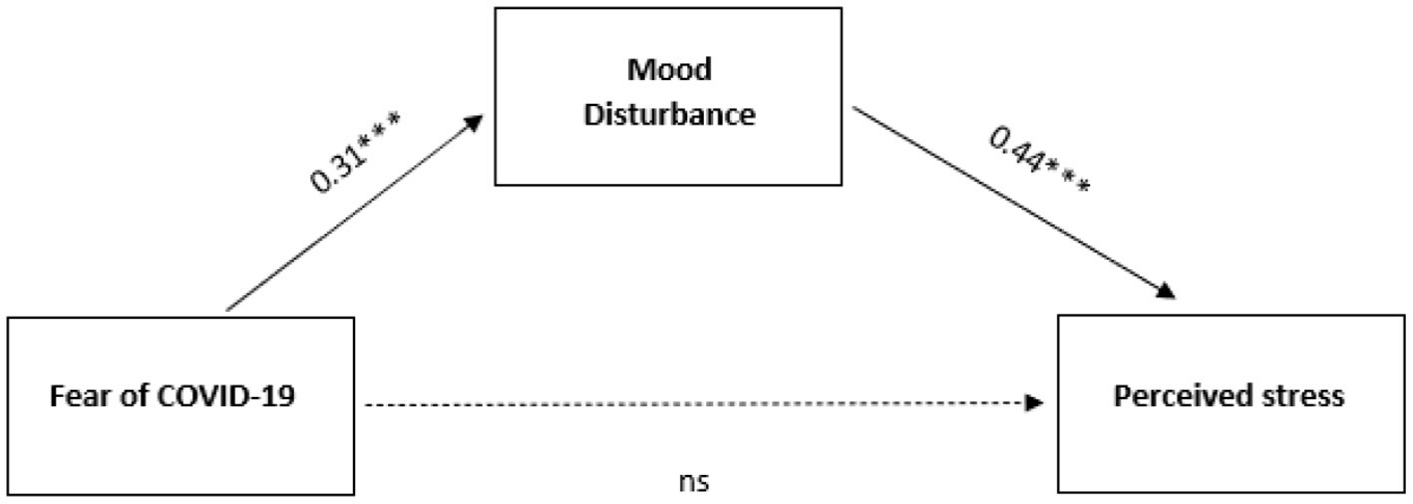
Tested model with Fear of COVID-19 as predictor, mood disturbance as mediator, and perceived stress as outcome for the whole sample.

The intercorrelation among variables was good, as shown in Table 2. The mediation model resulted significant for the indirect effect of Fear of Covid-19 on perceived stress [c] through mood disturbance (β = 0.14, *Z* = 2.80, and *p* = 0.005), but not for the direct effect [c'] (β = −0.04, *Z* = −0.48, and *p* = 0.63). Fear of COVID-19 was related to mood disturbance [a] (β = 0.31, *Z* = 3.45, and *p* < 0.001), and mood disturbance impacted the perceived stress [b] (β = 0.44, *Z* = 4.81, and *p* < 0.001; Figure 2). Therefore, apart from the stress due to the competition, it can be stated that the fear of COVID-19 generated mood disorders in the athletes, which in turns enhanced the degree of stress.

**Table 2:** Correlation among stress, mood disturbance, and fear of COVID-19 for the total sample.

	**(1)**	**(2)**	**(3)**
Stress (1)	1		
Mood disturbance (2)	0.43^***^	1	
Fear of COVID (3)	0.07	0.32^***^	1

*** *p < 0.001*.

## Discussion

The current paper aimed to examine the psychological profiles connected to the COVID-19 pandemic of the athletes competing in the World Sambo Championship, held in Novi Sad from the 6th to the 8th of November. Specifically, we hypothesized that the perceived stress was partially predicted by the fear of the pandemic through the mood disturbance resulting from this fear. We also elaborated on gender differences for the aforementioned variables, hypothesizing that female athletes should have displayed higher levels of Fear of COVID-19, higher mood disturbance, higher stress and worse sleep quality.

Acquired data showed that the Fear of COVID-19 actually produced mood disturbances which were reflected in a higher perception of stress, but the fear of COVID-19 did not predict directly the perceived stress. The gender comparison resulted in higher perception of stress and daytime disfunction in relation to sleep in females. No other gender differences appeared. It should be noted that nearly 90% of sambo athletes reported engaging in rapid weight loss (Drid et al., 2021), a practice that is defined as a ~5% weight loss achieved over 5–7 days, which can certainly contribute to overall stress as shown in previous studies (Franchini et al., 2012).

The researches available on the topic have shown that the exceptional circumstances of COVID-19 pandemic resulted in worsened mental health (O'Connor et al., 2020). Indeed, a study of Bakioglu et al. (2020) found that the Fear of COVID-19 directly influenced depression, anxiety, and stress, which in turn resulted in a decrease in positive thinking.

Another study showed that severe restrictions introduced during the pandemic had a direct impact on mood regulation, with high scores for tension, depression, anger, fatigue, and confusion (Terry et al., 2020). Namely, in the case of athletes, the COVID-19 national governments restrictions significantly increased their uncertainty about their careers and their future (Wilson et al., 2020), and preoccupation with training and physical shape (Schinke et al., 2020).

In the current study, the increase in negative mood affects in response to these restrictions has increased the athletes' perceived stress. Considering gender differences, females reported higher stress levels than males which is in alignment with findings of Di Fronso et al. (2020) who highlighted the negative effects of COVID-19 pandemic on athletes' perception of stress, confirming that women were more sensitive to this detrimental effect. Furthermore, the authors proposed two main explanations to be attributed to this diverse perception: first, females appear to ruminate on circumstances more than males (Nolen-Hoeksema and Jackson, 2001); second, during the pandemic, the uncertainty of female athletes' career and of their economic stability could have determined a bigger amount of perceived stress (Di Fronso et al., 2020).

Moreover, during the restrictions, other studies investigating the everyday life behavior found prolonged sleeping time (Pfefferbaum and North, 2020; Pišot et al., 2020). In addition, sleeping quality is an aspect that directly impacts the athletic performance, since it is an important component of recovery from training (Leeder et al., 2012). Indeed, athletes usually sleep worse before an important competition (Juliff et al., 2015). Our sample did not show any particular problem with respect to sleep quality and quantity. Regarding gender differences, they only appeared when it comes to daytime dysfunctions, where females obtained higher scores than males. Overall (regardless of the pandemic), daytime dysfunction problems are more common in females than males, probably because they are more involved in caregiving activities (Song et al., 2018).

The current study shows up with some limitations: first, the study has a correlational nature, therefore we do not know how this stress impacted the athletes' performance during the championship. Moreover, it would be interesting to examinate the intercorrelations among subscales, which was not performed due to reduced sample size. Therefore, it was impossible to perform two different models for males and females. Finally, the results are strictly connected to the pandemic situation and in a special condition (“the bubble”) that is an exceptional case, thus the generalizability of the results is limited. However, some studies outline certain benefits that “bubble” environment can produce. In particular, a study of McHill and Chinoy (2020) examined the effects of “bubble” conditions on performance during National Basketball Association (NBA) finals whereby athletes did not have to travel across different time zones and upset their circadian rhythm which can lead to sleep loss and fatigue, further negatively affecting athlete's health, performance, recovery, and mood (Leatherwood and Dragoo, 2013).

## Conclusion

However, the findings of the current study are useful to understand the psychological profile of athletes competing in a “bubble” conditions during the COVID-19 pandemic, characterized by a medium increase in stress levels in response to the mood oscillations generated by the pandemic. Competing in a similar situation is apparently safe, both for the limited risk of contagion and the psychological impact of the pandemic restrictions upon athletes' psychology.

## Data Availability Statement

The raw data supporting the conclusions of this article will be made available by the authors, without undue reservation.

## Ethics Statement

The studies involving human participants were reviewed and approved by Institutional Review Committee of the University of Novi Sad (Ref. No. 46-06-02/2020-1). The patients/participants provided their written informed consent to participate in this study.

## Author Contributions

AG, AB, NL, and PD wrote the article. AG, TT, AB, NL, FF, RR, SE, ST, NM, and PD designed the study, analyzed the data, discussed the results, reviewed, and approved the article. All authors contributed to the article and approved the submitted version.

## Conflict of Interest

The authors declare that the research was conducted in the absence of any commercial or financial relationships that could be construed as a potential conflict of interest.
