# Supplementary material for: Living in the “Bubble”: Athletes' Psychological Profile During the Sambo World Championship
Source: Front Psychol. 2021 May 26;12:657652. doi: 10.3389/fpsyg.2021.657652 (PMC8187578; doi:10.3389/fpsyg.2021.657652)
Supplement: Supplementary file 1 [file Data_Sheet_1.PDF]

HEALTH MEASURES DURING THE WORLD SAMBO CHAMPIONSHIPS 2020

This document has the goal to inform, guide, and ensure maximal safety prior and during the World SAMBO Championships 2020 in Serbia, Novi Sad from 4th of November to 9th of November 2020. It has been created following the World Health Organisation (WHO) recommendations, International Olympic Committee recommendation, International Labour Organisation (ILO) and with the participation of the FIAS Medical and Anti-Doping Commission as well as SAMBO Federation of Serbia - the organisers of the event.

1. SAFETY MEASURES - GENERAL INFORMATION

- ✓ **NEGATIVE PCR TEST -** Each delegation member is allowed to enter the Republic of Serbia only with a negative PCR test conducted within last 72 hours, issued by the approved national laboratory of the respective country. Based on the current pandemic situation, each delegation member entering to the Republic of Serbia from following countries: Croatia, Romania, Bulgaria and Republic of North Macedonia is obligatory to have a negative PCR test conducted within last 48 hours.

- ✓ **MANDATORY FACE MASKS -** Each national team and associated personnel are obliged to have a sufficient source of face masks. Mask-wearing is mandatory for everyone from the national team, except for athletes during the training or competition.

- ✓ **REGULAR TEMPERATURE CHECK -** Athletes are obliged to fill out form that they will receive, where body temperature will be inserted. The body temperature will be measured daily, in the morning (07:00-09:00) and in the evening (19:00-21:00). The completed form will be shared with the medical staff.

- ✓ **IN CASE OF SYMPTOMS -** In case a COVID-19-like symptom is detected, national team representatives will report this immediately to organisers of the championship Mrs. Tatjana Trivic – ttrivic@yahoo.com , +381645883545 . Potentially infected person will then undergo medical check with specialist Mr. Prof. Miodrag Drapsin, PhD, MD - minja.drapsin@gmail.com , +381655549563

- ✓ **BEHAVIOUR OF ACCREDITED PARTICIPANTS -** It is necessary to follow strict safety measures and respecting the approved standards: such as social distancing, mask-wearing and hand hygiene. Contact between athletes should be at minimum where only necessary team duties would be carried out. Going outside of "the bubble" will be prohibited. Spectators will be not permitted to enter the competition venue.

- ✓ **MEDICAL PRE-PARTICIPATION SCREENING -** Medical screening will be conducted by the official medical staff. Screening will be organized in such a way where only categories scheduled for that particular day are able to undergo screening. After FIAS standardized medical screening, body temperature will also be measured. If a participant has a body temperature higher than 38 degrees Celsius, he/she will be then proceeding the medical check with Mr. Prof. Miodrag Drapsin, PhD, MD - minja.drapsin@gmail.com , +381655549563.

- ✓ **INSURANCE** - It is the responsibility of the National Federation to ensure that in addition to their normal insurance they have sufficient insurance in place to cover any COVID-19-related costs. The organiser of the event and the FIAS accept no liability for any claims relating to cancellation of the event due to COVID-19 or medical costs for any COVID-19 related illness that may affect a National Federation delegation member during the event. The organiser of the event does not cover any other additional costs related to COVID-19 illness.

2. SAFETY MEASURES - DURING THE EVENT

- ✓ **ACCREDITATION -** Accreditation will take place in the official hotel. The organising committee will settle the exact schedule of accreditation and the team shall strictly respect the order. Disinfection fluids as well as proper sanitary materials will be provided. A maximum of two (2) representatives of the respective national team with all collected documents (passport, prove of payments, licences) will be allowed to proceed the accreditation process.

- ✓ **ACCOMODATION -** All teams will be accommodated at the hotel rooms where sufficient supplies of disinfection material will be provided. Athletes' nutrition will be in accordance with physical, religious and other dietary needs following the FIAS standards.

- ✓ **TRANSPORTATION-** Transportation of the athletes and the staff will be done in separate buses. Disinfection of those buses will be conducted twice per day. First one will be after athletes and

staff are transported and the second one will be done after daily duties are completed. Only delegation member is allowed to be transported in the buses where mask-wearing will be mandatory.

- ✓ **MEALS -** At the restaurant entrance disinfection fluids will be provided. Appropriate distance ideally 1.5 meter shall be maintained at the restaurant tables.

- ✓ **TRAINING PROCEDURE -** Training process will take place in alignment with national team plans in separate training facilities, as well as in the training arena. Mats can only be used by the athletes who will have to go through specialised disinfection check points. Coaches and medical staff can also be allowed to access mats.

- ✓ **MEDICAL CARE -** Medical care will be conducted in accordance with FIAS Medical Rules. Each medical team member will wear face mask, while during the interventions, glove-wearing will be mandatory, which will be disposed after the use. A separate medical care room will be dedicated to infectology due to the ongoing pandemic. This room will be clearly marked. All participants having COVID-19-like symptoms will be screened here. If needed, testing samples will be collected here and further analysed in the accredited institutions.

- ✓ **SOCIAL DISTANCING –** Physical dictating at least 1.5 meter must be respected all the time during the event. The exception in only given to athletes and referees during their bouts.

- ✓ **MAT HYGIENE –** All athletes shall pass disinfection checkpoints where shoes will be disinfected. Mat cleaning will take place right before the competition starts and any time needed during the competition as said in FIAS Medical Rules. Disinfection fluids will not harm skin of the participants.

- ✓ **EQUPIMENT SHARING –** Sharing any kind of equipment with other athletes such are SAMBO uniform, gloves, helmet, shoes are prohibited during the World SAMBO Championships 2020. FIAS will not provide to the athletes any spare uniform, gloves, helmet, shoes at this event.

- ✓ **DOPING TEST –** Doping tests will be conducted under FIAS Anti-Doping Rules and current WADA standards. All necessary sanitary measures will apply in order to avoid any risks and testing will be proceed following the latest WADA / FIAS safety recommendation.

- ✓ **VOLUNTEERS -** The entire organising committee staff who have contact with athletes have to prove negative PCR test up to 72 hours prior to the championship beginning.

The organizers of the competition, the chief referee of the competition, the medical delegate and the medical teams serving the tournament are responsible for coordinating and controlling the implementation of these measures and requirements.

These requirements are mandatory for all participants and organizers of sambo competitions. Failure to comply with the requirements may be a reason for disqualification of the participants of the competition.

The organising committee of the World SAMBO Championships 2020 as well as FIAS reserve the right to change above safety measurement based on current epidemic situation in Serbia.

Document approved by FIAS Medical and Anti-Doping Commission on 14th of October 2020.
